# Light-emitting diode therapy protects against ventricular arrhythmias by neuro-immune modulation in myocardial ischemia and reperfusion rat model

**DOI:** 10.1186/s12974-019-1513-5

**Published:** 2019-07-08

**Authors:** Songyun Wang, Lin Wu, Xuemeng Li, Binxun Li, Yi Zhai, Dongdong Zhao, Hong Jiang

**Affiliations:** 1 Department of Cardiology, Renmin Hospital of Wuhan University, Cardiovascular Research Institute of Wuhan University, Wuhan, 430060 Hubei People’s Republic of China; 2 Department of Cardiology, Shanghai Tenth People’s Hospital, Tongji University School of Medicine, Shanghai, 200072 People’s Republic of China

**Keywords:** Light-emitting diode, Myocardial ischemia-reperfusion, Ventricular arrhythmia, Microglia, Left stellate ganglion

## Abstract

**Background:**

Sympathetic overactivation and inflammation are two major mediators to post-myocardial ischemia-reperfusion (I/R)-induced ventricular arrhythmia (VA). The vicious cycle between microglia and sympathetic activation plays an important role in sympathetic hyperactivity related to cardiovascular diseases. Recently, studies have shown that microglial activation might be attenuated by light-emitting diode (LED) therapy. Therefore, we hypothesized that LED therapy might protect against myocardial I/R-induced VAs by attenuating microglial and sympathetic activation.

**Methods:**

Thirty-six male anesthetized rats were randomized into four groups: control group (*n* = 6), LED group (*n* = 6), I/R group (*n* = 12), and LED+I/R group (*n* = 12). I/R was generated by left anterior descending artery occlusion for 30 min followed by 3 h reperfusion. ECG and left stellate ganglion (LSG) neural activity were recorded continuously. After 3 h reperfusion, a programmed stimulation protocol was conducted to test the inducibility of VA. Furthermore, we extracted the brain tissue to examine the microglial activation, and the peri-ischemic myocardium to examine the expression of NGF and inflammatory cytokines (IL-1β, IL-18, IL-6, and TNF-α).

**Results:**

As compared to the I/R group, LED illumination significantly inhibited the LSG neural activity (*P* < 0.01) and reduced the inducibility of VAs (arrhythmia score 4.417 ± 0.358 vs. 3 ± 0.3257, *P* < 0.01) in the LED+I/R group. Furthermore, LED significantly attenuated microglial activation and downregulated the expression of inflammatory cytokines and NGF in the peri-infarct myocardium.

**Conclusions:**

LED therapy may protect against myocardial I/R-induced VAs by central and peripheral neuro-immune regulation.

## Introduction

Restoring blood flow to the ischemic myocardium by primary percutaneous coronary intervention or thrombolytic therapy is the most effective strategy for improving the patients’ clinical outcome after acute myocardial infarction (AMI). However, myocardial ischemia-reperfusion (I/R) injury, including I/R-induced ventricular arrhythmia (VA), remains a life-threatening complication and a neglected therapeutic target [1, 2]. Numerous previous studies have demonstrated that sympathetic overactivation [3] and inflammation [4] are two important pathophysiological mediators to myocardial I/R injury and VAs. However, there is still no effective therapy for preventing myocardial I/R injury and the occurrence of VAs.

It has been reported that the microglial activation in the hypothalamic paraventricular nucleus (PVN), indicating inflammation in this nucleus, was involved in many cardiovascular diseases related with neuro-immune dysfunction, such as AMI [5, 6] and hypertension [7]. Santisteban et al. [7] showed that there was a vicious cycle between sympathetic activation and microglial activation in the PVN in angiotensin-II-induced hypertension model. Furthermore, previous studies demonstrated that attenuating microglial activation with minocycline might decrease blood pressure, inhibit inflammatory response [7], and improve cardiac function post-AMI [8]. All these implicated that vicious cycle between sympathetic activation and microglial activation in the PVN might exist in neuro-immune activation related diseases and that microglial intervention might be a novel target for therapy.

Recently, phototherapy with light-emitting diode (LED) has been used for tissue repair with many protective effects, such as inhibiting inflammatory response and increasing collagen synthesis [9]. In an experimental ischemic stroke model, LED therapy attenuated neuroinflammatory responses and inhibited microglial activation, thus generating a smaller infarct size [10]. Therefore, we hypothesize that LED therapy might reduce VA inducibility by attenuating microglial and sympathetic activation after myocardial I/R. In the present study, we explored the effect of LED exposure to the body surface of PVN on VAs and neuro-immune activation in myocardial I/R rat model.

## Methods

### Animal preparation

Thirty-six Sprague-Dawley rats weighing 250–300 g were included in this study. Animals were housed in a specific pathogen-free room, with temperature (20–24 °C) and humidity (60–70%) and free access to standard rat chow and water. All study protocols were approved by the Animal Ethics Committee of Wuhan University under approval number 2015-0027 and followed the guidelines outlined by the Care and Use of Laboratory Animals of the National Institutes of Health. Rats were anesthetized by intraperitoneal injection of sodium pentobarbital (40 mg/kg) and then maintained with positive pressure ventilation at 70 breaths/min using a ventilator. The pain reflexes (estimated by pinching toes) during the surgery were observed to evaluate the anesthetic depth. Body surface electrocardiogram (ECG) was continuously recorded.

### LED

**Fig. 1:**
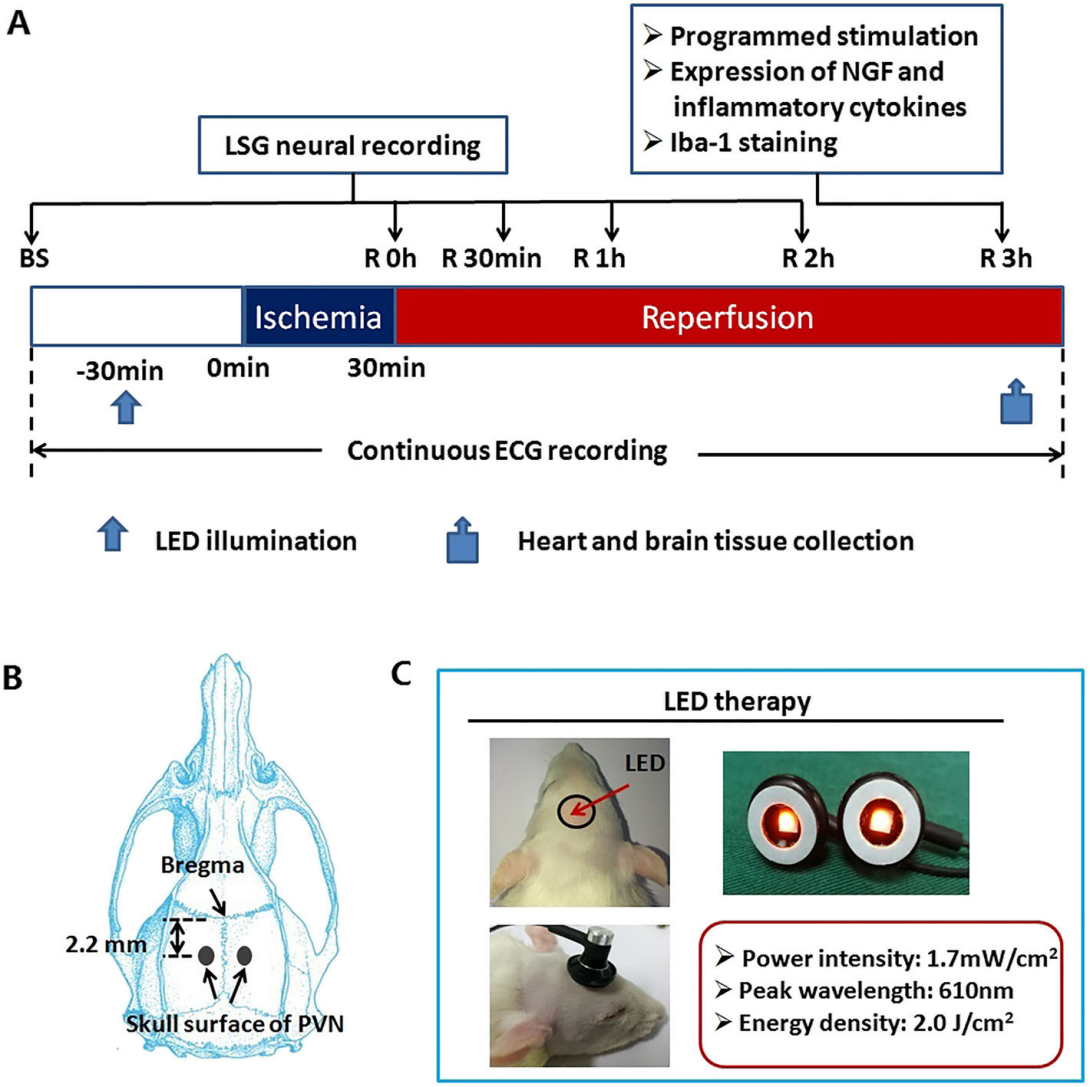
Experimental design flow chart of the LED+I/R group (**a**) and schematic representation of the skull surface of the hypothalamic PVN and technical characteristics of LED therapy (**b**, **c**). The I/R model was generated by left anterior descending artery occlusion for 30 min followed by 3 h reperfusion. LED illumination was performed from 30 min before ischemia to the end of the experiment in the LED+I/R group. The experimental procedure of I/R group (with sham LED therapy), LED group (with sham I/R operation), and control group (with sham LED therapy and sham I/R operation) was similar to the LED+I/R group. ECG, electrocardiogram; I/R, ischemia-reperfusion; LED, light-emitting diodes; LSG, left stellate ganglion; NGF, nerve growth factor; PVN, paraventricular nucleus

An LED light source (610 nm, Conver-gence Technology, Wuhan, China) was delivered at the skull surface of the hypothalamic PVN by adhesive tape for stability (Fig. 1c). The position of hypothalamic PVN was determined from the rat brain atlas of Paxinos and Watson, 2.2 mm posterior and 1.9 mm lateral to bregma and 7.3 mm below the skull surface [11] (Fig. 1b). For the LED and LED+I/R group, the rats received continuous LED therapy (peak wavelength, 610 nm; power intensity, 1.7 mW/cm^2^; energy density, 2.0 J/cm^2^) from 30 min before left anterior descending artery (LAD) occlusion to the end of the experiment (Fig. 1a). For the control group and I/R group, the rats received sham LED therapy.

### Myocardial ischemia and reperfusion model

A left thoracotomy was performed in the 4th intercostal space, the pericardium incised, and the heart exposed. In the I/R group and LED+I/R group, myocardial I/R model was induced by ligation of the LAD with a 6-0 silk thread and a piece of polyethylene tube for 30 min, and then loosening the silk thread for reperfusion for 3 h [12]. Ischemia was confirmed by an ST elevation on the ECG and the change in color of myocardial tissues of the ischemic area. A similar sham operation was conducted on the control and LED group without ligation of the LAD.

### Measurement of the left stellate ganglion (LSG) neural activity

Neural activity from LSG was recorded for 1 min. A tungsten-coated microelectrode was inserted into the fascia of the LSG, and a ground lead was connected to the chest wall. LSG-generated electrical signals were recorded with a Power Lab data acquisition system (8/35, AD Instruments, New South Wales, Australia) and an amplifier (DP-304, Warner Instruments, Hamden, CT). The bandpass filters were set at 300Hz to 1 kHz and the amplification range was 30 to 50 times. Neural activity, characterized by frequency and amplitude, was defined as deflections with a signal-to-noise ratio > 3:1. Both the amplitude and the frequency were manually determined as described in our previous studies [13].

### Programmed electrophysiological stimulation

To test ventricular arrhythmia (VA) inducibility, each heart was paced from the left ventricle using a programmed stimulation protocol. The stimulation was performed at eight beats of a cycle length of 120 ms (S1), followed by one to three extra-stimulus (S2, S3, and S4) at shorter coupling intervals. The end point of ventricular pacing was the induction of VAs. VAs including ventricular tachycardia (VT) and ventricular fibrillation (VF) were considered sustained when it lasted > 15 beats. An arrhythmia scoring system was used: 0, non-inducible preparations; 1, nonsustained tachyarrhythmias induced with three extrastimuli; 2, sustained tachyarrhythmias induced with three extrastimuli; 3, nonsustained tachyarrhythmias induced with two extrastimuli; 4, sustained tachyarrhythmias induced with two extrastimuli; 5, nonsustained tachyarrhythmias induced with one extrastimulus; 6, sustained tachyarrhythmias induced with one extrastimulus; and 7, tachyarrhythmias induced during the S1 stimulation. If the heart stopped before the pacing, the arrhythmia score assigned to that heart was 8. When multiple forms of VAs occurred in one heart, the highest score was used. The experimental protocols were typically completed within 10 min [14].

### Western blot and RT-PCR

Proteins were extracted from the frozen peri-infarct cardiac tissues. Protein concentrations were determined and normalized using the Bicinchoninic Acid Protein Assay Kit (G2026, Servicebio). Proteins (40 μg) were separated by sodium dodecyl sulfate-polyacrylamide gel electrophoresis and transferred onto a PVDF membrane. The PVDF membrane was blocked at room temperature with 5% powdered milk and incubated with primary antibodies against nerve growth factor (NGF, 1:1000, RLT3114, RuiYingBao), TNF-α (1:1000, ab66579, abcam), IL-6, (1:500, sc-57315, Santa), IL-1β (1:1000, bs-0812R, Bioss), and IL-18 (1:1000,10663-1-AP, Wuhan Sanying) overnight at 4 °C. Secondary antibodies were incubated with the membranes for 1.5 h at room temperature. GAPDH (1:25000, GB12002, Servicebio) was used to ensure equivalent protein loading.

To assess the mRNA expression levels of inflammatory cytokines in the peri-infarct myocardium, RNA was isolated using Trizol Reagent (G3013, Servicebio), and reverse-transcribed to complementary DNA using PrimeScript RT reagent Kit (#K1622, Fermentas) according to the manufacturer’s protocol. Then, RT-PCR was performed in a 20-μl reaction system containing complementary DNA, forward primer, reverse primer, and FastStart Universal SYBR Green Master (04913914001, Roche). The sequences of the inflammatory cytokines and GAPDH were shown in Table 1. We calculated the relative level of mRNA expression using the 2^−ΔΔCt^ method, normalized relative to GAPDH.

**Table 1:** Primer sequences and amplicon sizes of genes validated by Taqman RT-PCR

Gene name	Accession no.	Primer sequence	Amplicon size, bp
IL-1β	NM_031512.2	F: 5′-TGACCTGTTCTTTGAGGCTGAC-3′ R: 5′-CATCATCCCACGAGTCACAGAG-3′	272
IL-18	NM_019165.1	F: 5′-TCAGACCACTTTGGCAGACTTC-3′ R: 5′-GATTCGTTGGCTGTTCGGTC-3′	134
IL-6	R-IL6(RZ)-S R-IL6(RZ)-A	F: 5′-AGGATACCACCCACAACAGACC-3′ R: 5′-TTGCCATTGCACAACTCTTTTC-3′	109
TNF-α	R-TNF-α-S R-TNF-α-A	F: 5′-CCAGGTTCTCTTCAAGGGACAA-3′ R: 5′-GGTATGAAATGGCAAATCGGCT-3′	80
GAPDH	NM_017008.4	F: 5′-TCAGACCACTTTGGCAGACTTC-3′ R: 5′-TCAGACCACTTTGGCAGACTTC-3′	138

### Immunofluorescence staining

At the end of the experiment, hypothalamic PVN tissues of rats in each group were obtained for immunofluorescence staining. Tissues were sectioned into 5-μm slices and blocked with 5% normal fetal bovine serum in PBS for 2 h. Thereafter, the slices were incubated with primary antibody against Iba-1 (1:200, GB12105, Servicebio) overnight at 4 °C. The slices were then rinsed with PBS and incubated with Cy3-labelled secondary antibody (1:300, Servicebio) in the dark at room temperature for 60 min. To determine the microglial morphology, the staining imaging was observed with a fluorescence microscope and handled by Image-Pro Plus 6.0 software (Media Cybernetics, Rockville, MD, USA). Three microglial cells per brain slice, three brain slices per animal were measured from the hypothalamic PVN. The parameters of microglial morphology include soma area, processes length, and the number of primary branch projection (ramification). The number of Iba-1-positive cells was also calculated.

### Statistical analysis

Continuous variables were expressed as the means ± SEM. The changes in parameters were evaluated by *t* test and analysis of variance for comparisons. All the data was analyzed by GraphPad Prism 7.0 software (GraphPad Software, Inc., San Diego, CA), and *P* < 0.05 was considered as statistically significant.

### Study protocol

Animals were randomly divided into control group (*n* = 6), LED group (*n* = 6), I/R group (*n* = 12), and LED+I/R group (*n* = 12). The LED (2.0 J/cm^2^, 610 nm) device was located at the skull surface of hypothalamic PVN through scalp and skull from 30 min before ischemia to the end of the experiment. As shown in Fig. 1a, the LSG neural activity was measured at baseline, 30 min after ischemia, and 30 min, 1 h, and 2 h after reperfusion. ECG was recorded continuously and a programmed stimulation protocol was conducted to test the vulnerability of VA at 3 h after myocardial reperfusion. Furthermore, we extracted the heart to examine the expression of NGF and inflammatory cytokines (IL-1β, IL-18, IL-6, and TNF-α) and the brain for Iba-1 immunofluorescence staining.

## Results

### LED significantly inhibited the LSG neural activity

**Fig. 2:**
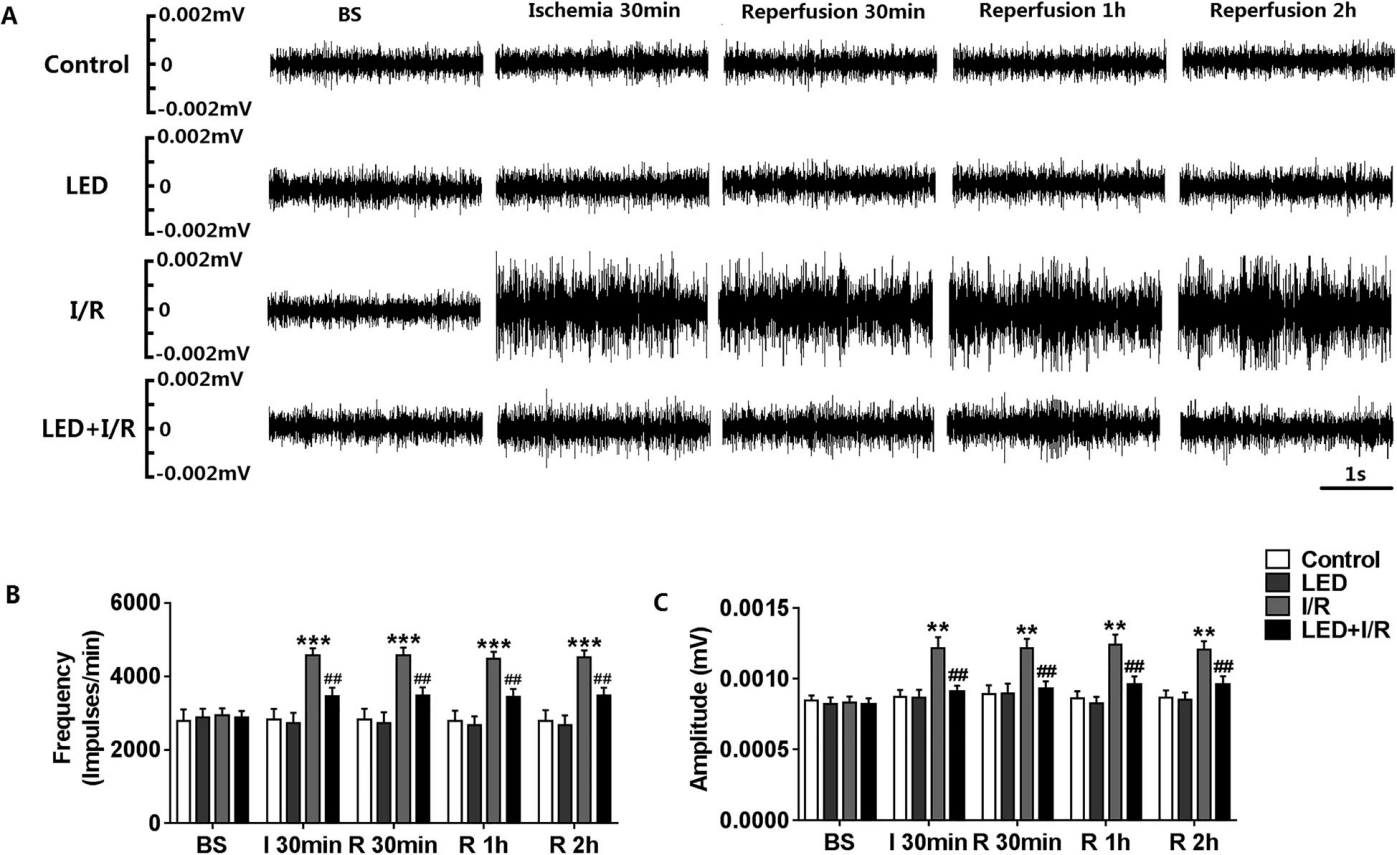
Effects of LED on LSG neural activity. **a** Representative examples of LSG neural activity in the control group, I/R group, LED group, and LED+I/R group. **b**, **c** Quantitative analysis of LSG neural activity in 4 groups. LED illumination significantly decreased the ischemia- and reperfusion-induced increase of amplitude and frequency of LSG neural activity. BS, baseline; LED, light-emitting diode; I, ischemia; R, reperfusion. **P < 0.01 and ***P < 0.001 as compared to the control group; ^##^*P* < 0.01 as compared to the I/R group

The representative examples of LSG neural activity recordings at different points in 4 groups were shown in Fig. 2a. As compared to the control group, there was a significant increase in both the frequency and the amplitude (Fig. 2b, c) of LSG neural activity after I/R in the I/R group. The neural activity in the LED group showed little difference statistically compared to the control group (Fig. 2b, c). Furthermore, compared to the I/R group, the I/R-induced increase of LSG neural activity was significantly reduced by LED illumination in both frequency (for ischemia 30 min, 4563.67 ± 196.93 impulses/min vs. 3463.17 ± 223.10 impulses/min; for reperfusion 30 min, 4579.33 ± 198.55 impulses/min vs. 3483.00 ± 215.25 impulses/min, both *P* < 0.01, Fig. 2b) and amplitude (for ischemia 30 min, 0.0012 ± 0.00008 mV vs. 0.0009 ± 0.00004 mV; for reperfusion 30 min, 0.0012 ± 0.00007 mV vs. 0.0009 ± 0.00006 mV, both *P* < 0.01, Fig. 2c) in the LED+I/R group.

### LED significantly reduced the inducibility of VAs

**Fig. 3:**
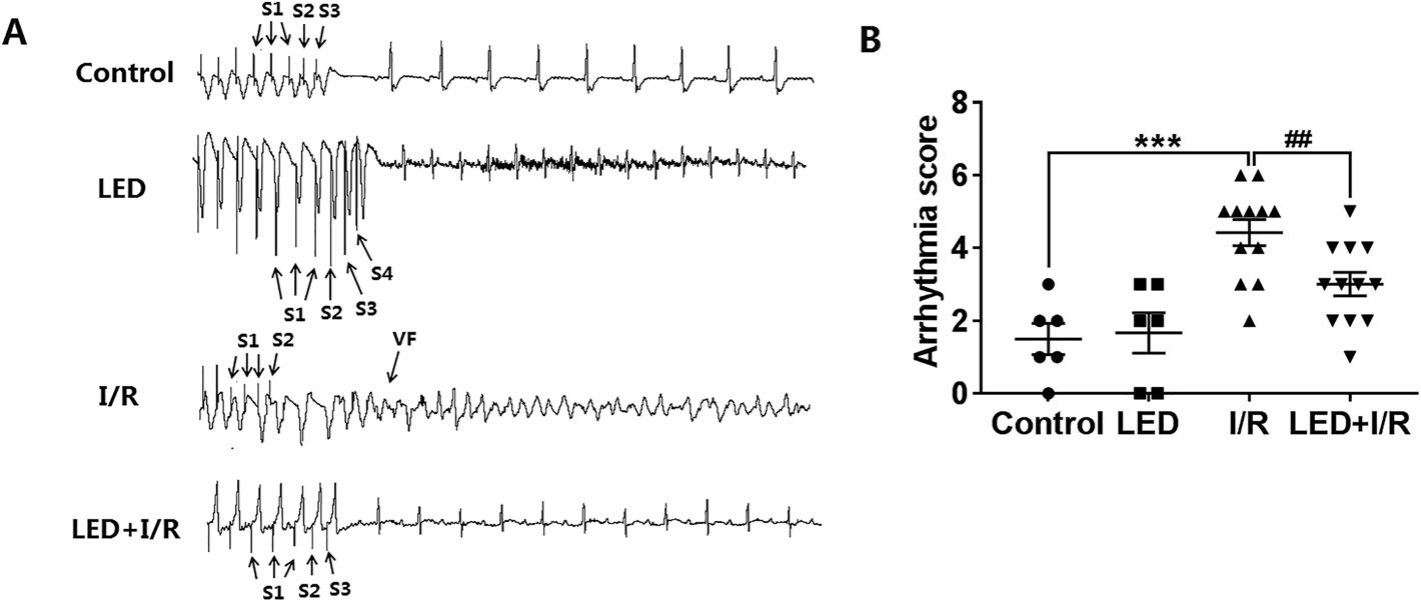
Effects of LED on ventricular arrhythmia inducibility. **a** Typical examples of programmed electrophysiological stimulation-induced VAs. **b** Arrhythmia score in 4 groups showed that LED illumination significantly decreased programmed stimulation-induced increased inducibility of VAs. I/R, ischemia-reperfusion; LED, light-emitting diodes; VA, ventricular arrhythmia; VF, ventricular fibrillation. ***P < 0.001 as compared to the control group; ##*P* < 0.01 as compared to the I/R group

Figure 3a showed the typical examples of programmed electrophysiological stimulation-induced VAs in 4 groups. Compared to the control group, the VA inducibility score was significantly increased in the I/R group (*P* < 0.001, Fig. 3b), whereas this was significantly reduced by LED illumination in the LED+I/R group (arrhythmia score, 4.42 ± 0.36 vs. 3.00 ± 0.33, *P* < 0.01, Fig. 3b).

### LED significantly attenuated microglial activation in the PVN

**Fig. 4:**
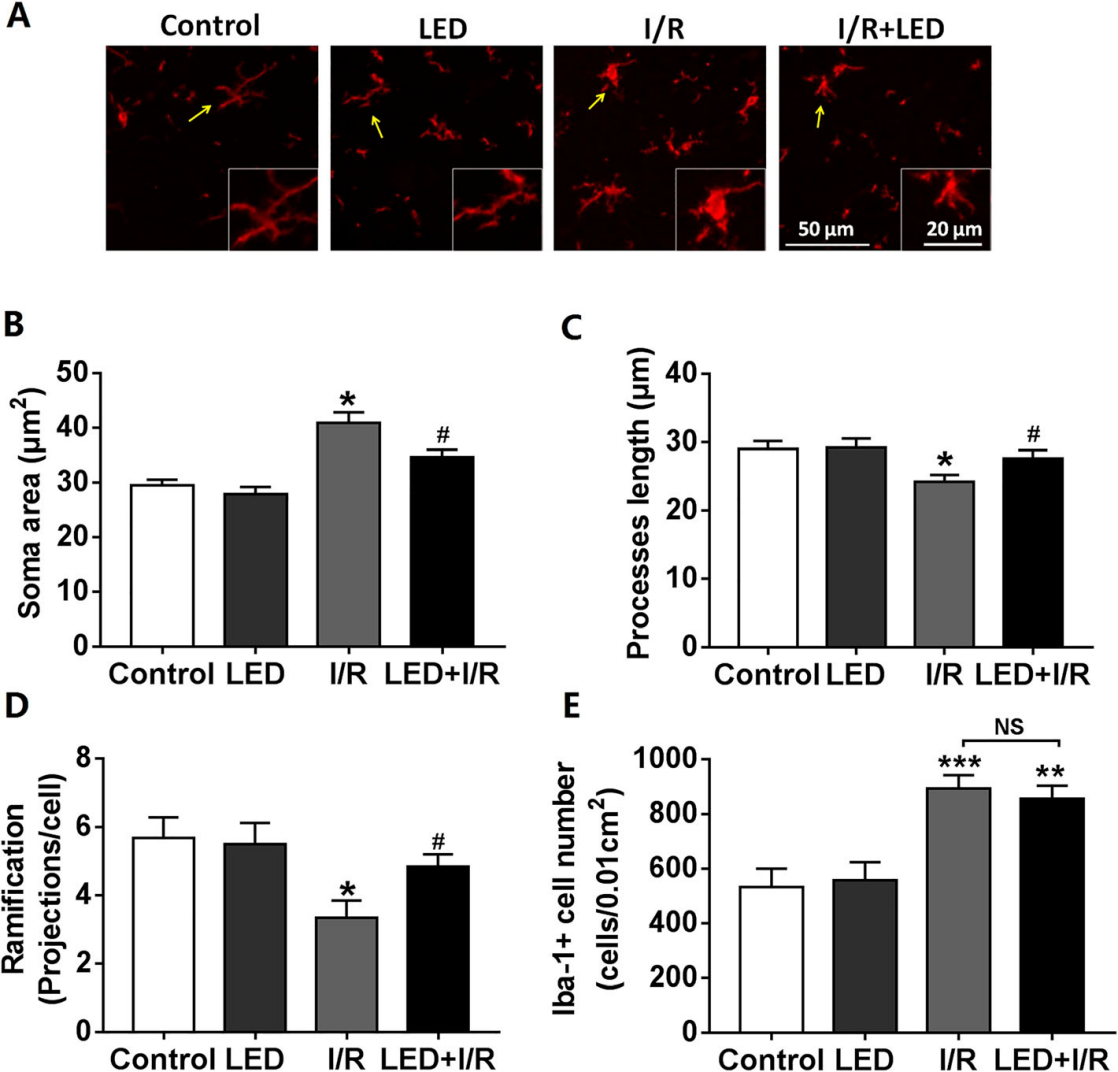
Effects of LED on microglial morphology. **a** Representative images of Iba-1 immunofluorescence staining in the PVN of rat brain in different groups. Scale bar = 50 μm. **b**–**e** Quantitative analysis of soma area, processes, projection of Iba-1-positive cell, and number of Iba-1-positive cell. I/R, ischemia/reperfusion; LED, light-emitting diode. *P < 0.05, **P < 0.01, and ***P < 0.001 as compared to the control group; ^**#**^*P* < 0.05 and ^**##**^*P* < 0.01 as compared to the I/R group; NS, *P* > 0.05

To examine the effect of myocardial I/R and LED therapy on the activation of microglia, immunofluorescence staining was used for Iba-1 in the PVN. The microglial morphology of Iba-1 immunofluorescent was demonstrated in Fig. 4a. Compared to the control group, the LED group showed no significant difference in microglial morphology and number (Fig 4a–e), and microglia in the I/R group had amoeboid phenotype as shown by the significantly increased soma size (Fig. 4b), decreased processes length (Fig. 4c), and decreased major projection (Fig. 4d). All these morphological changes indicated the activation of microglia [15], and they were significantly attenuated by LED illumination (Fig. 4b–d). Besides, compared to the control group, the number of Iba-1+ cells in PVN was increased in both the I/R group (*P* < 0.001) and the LED+I/R group (*P* < 0.01, Fig. 4e).

### LED significantly downregulated the expression of inflammatory cytokines and NGF in the myocardium

**Fig. 5:**
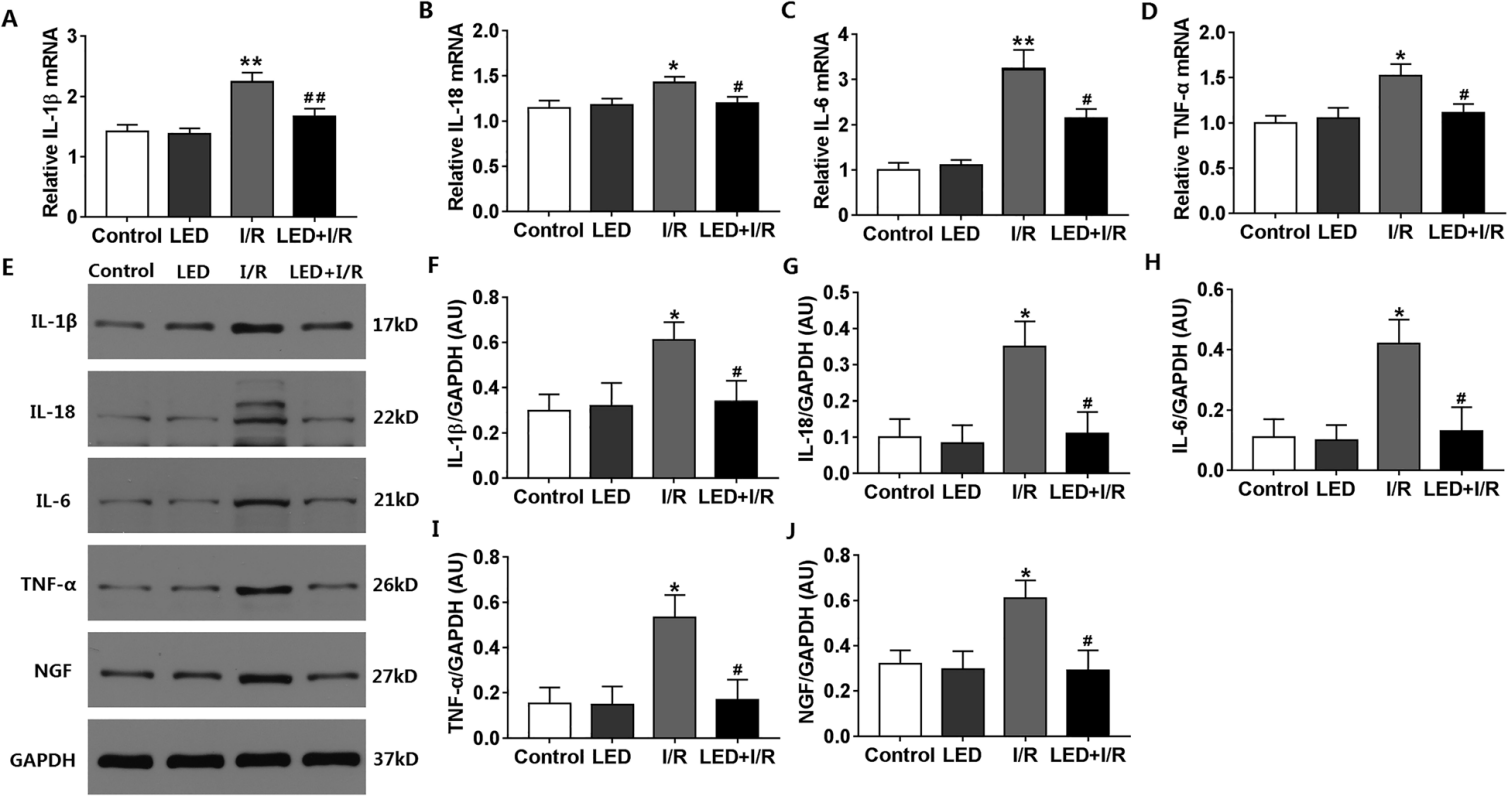
Effects of LED on the expression of inflammatory cytokines and NGF in the myocardium. **a**–**d** IL-1β, IL-18, IL-6, and TNF-α RNA expression in different groups. **e** Typical immunoblots of inflammatory cytokines and NGF expression. **f**–**j** Quantitative analysis of IL-1β, IL-18, IL-6, and TNF-α and NGF protein expression. I/R, ischemia/reperfusion; LED, light-emitting diode; NGF, nerve growth factor. *P < 0.05 and **P < 0.01 as compared to the control group; ^#^*P* < 0.05 and ^##^*P* < 0.01 as compared to the I/R group

RT-PCR was done to explore whether the inflammatory response was involved in the protective effect of LED illumination in the inducibility of VAs post-myocardial I/R. The study demonstrated that LED might significantly reduce myocardial I/R-induced increase of proinflammatory cytokines IL-1β (*P* < 0.01, Fig. 5a), IL-18 (*P* < 0.05, Fig. 5b), IL-6 (*P* < 0.05, Fig. 5c), and TNF-α (*P* < 0.05, Fig. 5d) mRNA expression. Figure 5e showed typical immunoblots images of the protein expression of various inflammatory cytokines and NGF. Compared to the control group, all the expression of these proteins was significantly upregulated in the I/R group and LED illumination significantly decreased these changes (Fig. 5f–j) in the LED+I/R group.

## Discussion

### Major findings

In the present study, we demonstrated the LED illumination to inhibit the SNA and inflammatory response and suppress myocardial I/R-induced VAs. As compared to the I/R group, LED illumination significantly reduced myocardial I/R-induced increase of VA inducibility (arrhythmia score). In peripheral tissues, LED illumination significantly attenuated the myocardial I/R-induced increase in both sympathetic nerve activity (LSG neural activity, expression of NGF) and proinflammatory cytokines (expression of IL-18, IL-1β, IL-6, and TNF-α). In the hypothalamic PVN of central tissues, microglial activation was significantly inhibited by LED illumination. All these results indicated that LED illumination might protect against myocardial I/R-induced VAs by attenuating SNA and inflammatory response both in central and peripheral tissues.

### Sympathetic overactivation, inflammation, and myocardial I/R-induced VAs

It is well established that the overactivation of the cardiac sympathetic nervous system, especially LSG, plays a crucial role in the occurrence of VAs and myocardial I/R injury [16, 17]. Inhibiting the LSG neural activity with LSG denervation and blockade has been applied to the treatment of VAs [16]. However, this may lead to some undesirable side effects, such as Horner’s syndrome and chest pain. Therefore, exploring a novel method is pressing. Hypothalamic PVN is an important site of the central nervous system which modulates the autonomic nervous system of cardiovascular diseases [18]. Besides, the previous study demonstrated that AMI might result in a significant increase of the sympathetic neuron activity in the PVN, thus promoting an elevation of LSG neural activity [19]. Furthermore, NGF, as a novel biomarker in myocardial infarction, plays a vital role in the differentiation, survival, and synaptic activity of sympathetic nerves after myocardial I/R [20]. Overexpression of NGF after myocardial I/R results in the sprouting of cardiac autonomic nerve, sympathetic hyper-innervation, and increase of SNA, which contributes to the incidence of VAs and sudden cardiac death [21, 22]. All these indicated that PVN might be a novel target for VAs therapy.

Another key mediator involved in myocardial I/R injury and VAs is the immune and inflammatory response [23, 24]. Previous studies have confirmed that myocardial I/R, in the acute phase, triggers the production and release of varieties of inflammatory chemokines and cytokines and generates a serial vicious immune response, which plays a crucial role in myocardial injury [24, 25]. In the first 3–12 h after myocardial ischemia, expression of IL-1β and IL-6 was significantly increased in both ischemia and non-ischemic myocardium [26]. Besides, inflammasome activation has been reported to result in infiltration of proinflammatory immunocytes and expression of various inflammatory cytokines in cardiomyocyte, such as IL-1β and IL-18 [25, 27]. Studies have also demonstrated that immune response is performed not only in peripheral tissues, such as plasma and myocardium, but also in the hypothalamic PVN of the central nervous system after AMI [28]. All these results indicate that inflammation is critically involved in the incidence of VAs during myocardial ischemia and reperfusion and that attenuating both peripheral and central inflammatory response may be a therapeutic method to reduce malignant VAs. In addition, the previous study has found that the increase of SNA may cause the activation of inflammasome and increase of IL-18 in the myocardium, thus exacerbating the inflammatory response and cardiac injuries [27]. Our present study also showed that myocardial I/R might result in a significant increase of microglial activation in the PVN. Furthermore, the LSG neural activity and expression of inflammatory cytokines were significantly increased in the I/R group. All these findings indicated that the increase of SNA and inflammatory response post-myocardial I/R might be the pathophysiological mechanisms of the occurrence of VAs.

### Microglia and sympathetic nervous system

Microglia, serving as primary mediators of the immune defense system of the central nervous system, activate and release proinflammatory mediators in pathological conditions [29], thus leading to neuronal dysfunction and cell death [30]. Previous studies have demonstrated that the microglial activation in the PVN plays an important role in cardiovascular diseases which are associated with neuro-immune dysfunction, such as AMI [5, 6] and hypertension [7]. Furthermore, due to various receptors for neurotransmitters expressed on the membrane of microglia and a close position between microglia and sympathetic neuron in the PVN, microglia are allowed to perceive sympathetic neuronal signals and have constant communication with sympathetic neurons [31]. The previous study showed that intracerebroventricular administration of IL-1β might result in microglial activation and increase arterial blood pressure, heart rate, and renal SNA [32]. Furthermore, research showed that there was a vicious cycle between the microglial activation and sympathetic activation in angiotensin II-induced hypertension model [7]. Moreover, administration of minocycline, known to attenuate microglial activation, might decrease blood pressure, inhibit inflammatory response [7], and ameliorate cardiac function post-AMI [8]. In our study, the results showed that myocardial I/R might cause a significant increase of microglial and sympathetic activation, and LED illumination might reduce the inducibility of VAs by inhibiting these changes. All these studies suggest that microglial might be involved in the modulation of SNA and attenuating microglia activation might be a therapeutic method for VAs post-myocardial I/R.

### LED therapy for VAs post-myocardial I/R and neuro-immune regulation

**Fig. 6:**
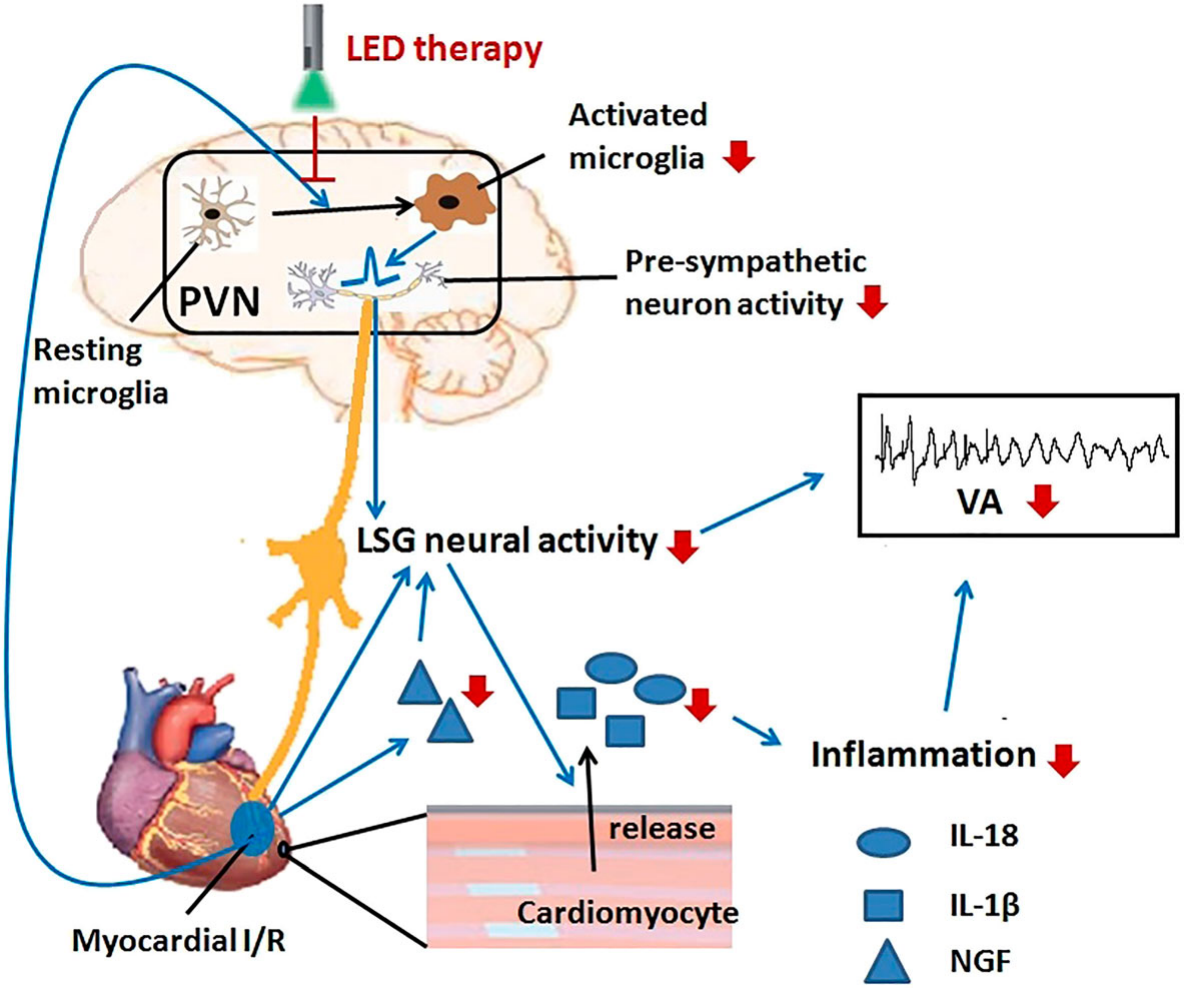
Proposed hypothesis for the therapeutic effect of LED on VAs in myocardial I/R. Myocardial I/R might cause a series of changes directly or indirectly: microglia activation in the PVN and increase of LSG neural activity and inflammatory response in the myocardium. LED therapy might inhibit the microglia activation induced by myocardial I/R, thus suppressing the sympathetic activation and inflammatory response. These changes help to protect against VAs. I/R, ischemia-reperfusion; LED, light-emitting diodes; LSG, left stellate ganglion; NGF, nerve growth factor; PVN, paraventricular nucleus; VA, ventricular arrhythmia

Photobiomodulation (PBM), using a wavelength ranging from 600 to 1000 nm light source such as LED or low-level laser therapy, has attracted attention for inducing a beneficial biological response in cells and tissues [33], like increasing ATP synthesis and fibroblast proliferation and inhibiting oxidative stress and inflammatory response [9]. Though few studies have evaluated the transmission rate of radiation in the skull, it is well acknowledged that a 600–800-nm radiation can penetrate approximately 1 cm into the skull [34], which provides a theoretical basis for our study. Recent studies have found that LED therapy might reduce neuroinflammatory response by attenuating microglial activation and neutrophil infiltration. LED therapy also downregulated the expression of proinflammatory cytokines IL-18 and IL-1β and attenuated the NLRP3 inflammasome in an ischemic stroke model [35]. The precise mechanisms underlying the effect of LED therapy on neuroinflammation has not been well-established yet, but LED can participate in various cellular responses because photons can be absorbed by the enzyme cytochrome c oxidase in the mitochondria [36]. It contributes to the elevation of ATP and reactive oxygen species, modulation of transcription factor, and activation or inhibition of downstream signaling pathway [37, 38]. For example, LED therapy might inhibit the neuroinflammatory response by suppressing TLR-2 levels, MAPK signaling, and NF-κB activation [35]. The previous studies showed that LED at shorter wavelengths (300–400 nm) might increase the expression of some proinflammatory cytokines [39, 40], but LED showed an anti-inflammatory effect at longer wavelengths (600–850 nm) [41, 42]. Therefore, the effects of LED on inflammatory response varied from the wavelengths of light. In LPS-activated microglia like BV-2 cells, PBM decreased the production of nitric oxide, downregulated the expression of inducible nitric oxide synthase, and attenuated inflammatory cytokine TNF-α [38]. Besides, PBM could also decrease activated microglial cytotoxicity and enhance the phagocytic activity [38]. In the present study, we have found that LED (610 nm) illumination significantly attenuates microglia activation in the PVN and significantly decreased the LSG neural activity and the expression of IL-1β, IL-18, IL-6, and TNF-α in the myocardium. All these results suggested that LED illumination of hypothalamic PVN might protect against VAs post-myocardial I/R by neuro-immune regulation (Fig. 6).

### Limitation

This study investigated the effect of LED illumination on VA inducibility post-myocardial I/R. However, there are still some limitations in the present study. First, the sympathetic nervous system might be affected by the anesthesia with pentobarbital, but this could be counteracted as all the groups are performed in the same way. Second, the LED device was so large that we could not guarantee the PVN-targeted stimulation and we were not sure whether LED might have an influence on the surrounding brain tissues. Third, we only investigate the effect of LED therapy on myocardial I/R-induced VAs in acute phase, and it was uncertain whether these anti-arrhythmia effects would continue to exist or how long these effects would persist in the chronic phase. Fourth, the study did not examine the phenotype of microglia in the PVN and the direct link between microglia and the central sympathetic neurons. Fifth, it was possible that the environmental light might have affected the results. However, this could be counteracted because all the experiments were conducted in the same environment.

### Clinical implication

In the present study, we have found that LED therapy might reduce VA inducibility post-myocardial I/R by modulating the sympathetic nervous system and inflammation. Unlike other interventions which have potentially deleterious serious side effects, LED therapy has gained more attraction because it is relatively noninvasive, affordable, compact, and has fewer side effects. Besides, LED generates negligible heat at the surrounding tissues of the device. In addition, longer red/near-infrared wavelengths are easier to penetrate tissues than shorter blue/green wavelengths; therefore, red and near-infrared lights are preferred clinically [35]. Since the light source is able to penetrate the skull and scalp, LED therapy might be a novel and noninvasive therapeutic method to protect against the VA post-myocardial I/R.

## Conclusions

This study has shown that LED therapy significantly attenuates myocardial I/R-induced increase in microglia activation, SNA, and proinflammatory cytokine expression, ultimately reducing the VA inducibility. Therefore, LED therapy might protect against VAs induced by myocardial I/R by neuro-immune modulation.

## Data Availability

The datasets supporting the conclusions of this article are included within the article.

## Abbreviations

AMI: Acute myocardial infarction
ECG: Electrocardiogram
I/R: Ischemia-reperfusion
LAD: Left anterior descending artery
LED: Light-emitting diode
LSG: Left stellate ganglion
NGF: Nerve growth factor
PBM: Photobiomodulation
PVN: Paraventricular nucleus
SNA: Sympathetic nerve activity
VA: Ventricular arrhythmia
VF: Ventricular fibrillation
VT: Ventricular tachycardia
